# Skin-to-Skin Contact at Birth for Very Preterm Infants and Mother-Infant Interaction Quality at 4 Months: A Secondary Analysis of the IPISTOSS Randomized Clinical Trial

**DOI:** 10.1001/jamanetworkopen.2023.44469

**Published:** 2023-11-30

**Authors:** Siri Lilliesköld, Karoline Lode-Kolz, Siren Rettedal, Johanna Lindstedt, Agnes Linnér, Hanne Markhus Pike, Sari Ahlqvist-Björkroth, Ulrika Ådén, Wibke Jonas

**Affiliations:** 1 Department of Women’s and Children’s Health, Karolinska Institutet, Stockholm, Sweden; 2 Department of Neonatology, Astrid Lindgren Children’s Hospital, Karolinska University Hospital, Stockholm, Sweden; 3 Faculty of Health Sciences, University of Stavanger, Stavanger, Norway; 4 Department of Clinical Neurophysiology, Stavanger University Hospital, Stavanger, Norway; 5 Department of Paediatrics, Stavanger University Hospital, Stavanger, Norway; 6 Department of Research, Stavanger University Hospital, Stavanger, Norway; 7 Department of Psychology and Speech-Language Pathology, University of Turku, Turku, Finland; 8 Department of Clinical Science, Intervention and Technology, Karolinska Institutet, Stockholm, Sweden; 9 Department of Clinical Medicine, University of Turku, Turku, Finland; 10 Department of Biomedical and Clinical Sciences, Linköping University, Linköping, Sweden

## Abstract

**Importance:**

Good-quality parent-infant interactions have protective effects on infant socio-emotional and behavioral development. These interactions are especially critical for very preterm infants at risk of vulnerabilities related to immaturity. Skin-to-skin contact (SSC) has been found to improve mother–preterm infant interaction behaviors, but few studies exist regarding its benefits when initiated immediately after birth.

**Objective:**

To determine the effect of immediate SSC at birth for very preterm infants on mother-infant interaction quality at 4 months of corrected age.

**Design, Setting, and Participants:**

This secondary analysis used data from the Immediate Parent-Infant Skin-to-Skin Study (IPISTOSS), a randomized clinical trial conducted between April 1, 2018, and June 30, 2021, at 3 neonatal units in Sweden and Norway. Participants included very preterm infants (28-33 gestational weeks of age) and their parents. Four-month follow-up was concluded in December 2021. Data analyses were performed on March 16 and September 18, 2023.

**Intervention:**

Infants were allocated to standard incubator care or SSC with either parent initiated at birth and continued throughout the first 6 hours after birth.

**Main Outcomes and Measures:**

The primary outcome was mother-infant interaction quality as measured with the Parent–Child Early Relational Assessment (PCERA), based on video recordings of a 5-minute free-play situation with mother-infant dyads at 4 months of corrected age. A multilevel regression analysis was performed.

**Results:**

This analysis included 71 infants (31 twins [44%]) and 56 mothers. Infants had a mean (SD) gestational age of 31 weeks 3 (1.3) days, and more than half were male (42 [59%]); mothers had a mean (SD) age of 32 (4.9) years. There were 37 infants allocated to standard care and 34 to SSC with either parent after birth. During the first 6 hours after birth, fathers provided more SSC than mothers, with a median (IQR) of 3.25 (2.25-4.5) and 0.75 (0-2.5) hours, respectively. A statistically significant difference in 1 of 5 PCERA subscales (subscale 3: infant positive affect, communicative and social skills) was observed, with higher-quality mother-infant interaction in the SSC group at 4 months (Cohen *d* = 0.67 [95% CI, 0.17 to 1.17]; *P* = .01). This effect remained significant when adjusting for primiparity, child sex, and observation setting.

**Conclusions and Relevance:**

In this study of the effect of immediate parent-infant SSC after very preterm birth, SSC was beneficial for the mother-infant relationship. These findings suggest that immediate SSC should be supported in the clinical setting.

**Trial Registration:**

ClinicalTrials.gov Identifier: NCT03521310

## Key Points

**Question:**

Is skin-to-skin contact (SSC) between parents and their very preterm infants immediately after birth more effective than standard incubator care in enhancing mother-infant interaction at 4 months?

**Findings:**

In this secondary analysis of a randomized clinical trial including video recordings of 71 mother–very preterm infant dyads in interaction during free play, SSC provided by a parent during the first 6 hours after birth significantly enhanced child interactive behaviors and positive affect at 4 months.

**Meaning:**

These findings suggest that alongside necessary nursing and medical care, clinical practices should support the mother-infant relationship by promoting immediate SSC with a parent after birth.

## Teaser

This secondary analysis of the IPISTOSS randomized clinical trial examines the effect of immediate skin-to-skin contact at birth for very preterm infants on mother-infant interaction quality at 4 months of corrected infant age.

## Introduction

Skin-to-skin contact (SSC) is an evidence-based method of care that involves placing the naked infant on the parent’s bare chest. The SSC method is routinely practiced intermittently for preterm infants in the neonatal unit. Because of its multiple benefits, SSC between newborn infants and mothers provided in the immediate period after birth is considered the standard of care.^1,2,3,4^ For infants born preterm, the timing of SSC initiation can vary greatly.^5,6^ In a Swedish population-based register study, only a minority of infants born before gestational week 32 received SSC within their first day of life.^7^ Recent guidelines from the World Health Organization recommend immediate postnatal initiation of SSC for preterm or low-birth-weight infants,^8^ due to the increase in survival rates.^9^ Data on long-term developmental effects of immediate SSC for very preterm infants, such as the quality of the parent-infant relationship, are limited.

Good-quality early parent-infant interactions have protective effects on infant social-emotional, cognitive, and behavioral development^10,11^ and are critical for preterm infants with inherent vulnerabilities related to immaturity.^12,13^ Mother-infant interaction has been described as an act of reciprocity,^14^ in which both participants modify their behaviors depending on feedback provided by the other. It is often more difficult for parents to interpret and respond to the behavioral cues of preterm infants because these cues are weaker.^15^ Moreover, becoming a parent to a preterm infant can be stressful and has been linked to compromised psychological well-being in parents^16^ and reduced maternal sensitivity,^17^ which may affect interaction quality. Differences in dyadic interaction behaviors between mothers and preterm infants, compared with mother and full-term infant dyads, are most evident during the first 6 months of life.^18^ Early interventions that foster close parent-infant contact are therefore warranted.^19,20^

Skin-to-skin contact provides an environment that is sensitive to the infant’s needs and gives opportunities for emotionally supportive interactions that stimulate brain growth and development.^21,22^ When in SSC, parents can respond more promptly to infant cues, providing opportunities for co-regulation.^23^ In a systematic review,^24^ SSC improved parent–preterm infant interaction patterns, but the timing of SSC was not investigated. Indeed, the first hour or hours after birth have been described as an early sensitive period,^25,26^ characterized by neuroendocrine changes that provide a biological and behavioral basis for social interaction, bonding, and attachment.^27,28,29^ In term infants, 2 hours of SSC after birth positively influenced mother-infant interaction (maternal sensitivity, child self-regulation, and dyadic reciprocity) 1 year later.^30^ In preterm infants, SSC onset within the first 3 days favored the development of infant interactive behavior at 6 months,^31^ and SSC within the first day of life predicted mothers’ sensitivity to their infant.^32^ Very preterm infants placed in SSC with their mother for 60 minutes at 1 hour after birth demonstrated better responses during mother-infant interaction at 6 months compared with dyads with only visual contact.^33^ To our knowledge, no study has evaluated the effect of SSC with either parent immediately after birth on mother-infant interaction.

There seem to be critical time points after birth when SSC is especially important for the developing parent-infant relationship. Yet evidence remains scarce as to whether parent-infant SSC initiated immediately after birth for preterm infants is more beneficial than standard incubator care (with later initiation of SSC), in terms of supporting the mother-infant relationship. Thus, the main objective of this study was to determine the effect of immediate SSC at birth for very preterm infants on mother-infant interaction at 4 months of corrected infant age. An exploratory and secondary objective was to investigate whether the potential relationship between immediate SSC after birth and mother-infant interaction at 4 months would be mediated through accumulated SSC in the early postpartum period (within 72 hours and 8 days after birth). We hypothesized that SSC provided to the very preterm infant within the first hours after birth improves mother-infant interaction quality in infancy.

## Methods

### Study Design

This secondary analysis reports on a secondary outcome from the Immediate Parent-Infant Skin-to-Skin Study (IPISTOSS), a randomized clinical trial with 2 parallel, nonblinded groups conducted between April 1, 2018, and June 30, 2021.^34^ Electronic randomization was performed in uneven block sizes, with a 1:1 ratio, and stratified by site and gestation (28 weeks 0 days to 30 weeks 6 days and 31 weeks 0 days to 32 weeks 6 days). Ethical approval was obtained from the Swedish Ethical Review Authority and the Norwegian Regional Ethical Committee. Research staff informed parents meeting the IPISTOSS inclusion criteria, and written informed consent was obtained from both parents before birth. The study followed the Consolidated Standards of Reporting Trials (CONSORT) reporting guideline.

### Setting and Population

The IPISTOSS study was conducted at 2 neonatal units at Karolinska University Hospital in Stockholm, Sweden, and at the neonatal unit at Stavanger University Hospital in Stavanger, Norway. Screening was performed for women admitted to obstetric units with threatening preterm labor. This study included inborn infants (singletons or twins) with a gestational age of 28 weeks 0 days to 32 weeks 6 days, regardless of birth mode. Infants with congenital infection, major malformations, or other conditions deemed contraindicating to participation were excluded.

### Intervention and Procedure

The intervention consisted of SSC between either parent and their very preterm infant (or infants) initiated immediately after birth (SSC group) and continued throughout the first 6 hours after birth and was compared with conventional care in an incubator or cot (control group). Electronic randomization was performed by research staff when birth was imminent. Twins were allocated to the same study group. During the intervention period, only the place of care differed; all other monitoring, nursing, and medical care were identical in both groups, as previously described in the IPISTOSS research protocol in Supplement 1.^34^

#### Intervention

After vaginal birth, SSC was initiated immediately, or as soon as possible, on the mother’s chest, with positioning assisted by the neonatal team. The infant was cared for initially in the birth unit and later transferred to the neonatal unit while maintaining SSC with either parent. After cesarean delivery, SSC was initiated with the father until the mother could be transferred to the neonatal intensive care unit. Twins were either cared for with one parent each or placed together with one of the parents.

#### Conventional Care

Infants allocated to the control group were stabilized in a warmer (Resuscitaire; GE Healthcare) or in an incubator and then transported to the neonatal unit in an incubator. Intermittent SSC was initiated after the first 6 hours. Parents in the control group were allowed to stay at their infant’s bedside and were able to touch the infant in the incubator or cot.

### Follow-Up Visit at 4 Months

A follow-up visit was conducted at 4 months (±2 weeks) of corrected infant age in the clinic or at participant homes (in Sweden, 20 visits [57%] were conducted at participant homes due to hospital restriction policies during the COVID-19 pandemic). The visit was made in the morning at a time when the infant preferably had slept and was newly fed. Baseline data regarding maternal mental health (Edinburgh Postnatal Depression Scale^35^ and Spielberger State-Trait Anxiety Inventory^36^) and parental stress (Swedish Parenthood Stress Questionnaire^37^) were collected. Mother-infant interaction was video recorded according to a standard procedure.^38^

### Outcome Assessment

#### Duration of SSC

Duration of SSC (in hours per day) was recorded with the Parent-Infant Closeness Diary^39^ by research staff during the intervention and by parents during the first week after birth daily, overseen by research staff. With 15-minute accuracy, mothers and fathers drew separate timelines showing when they provided SSC.

#### Mother-Infant Interaction Quality

Mother-infant interaction quality was assessed with the Parent–Child Early Relational Assessment (PCERA)^38^ during video-recorded free play. Mothers were instructed to play with their infant as usual, with preselected toys available. The PCERA includes 29 parent items, 28 child items, and 8 dyadic items. Based on a 5-minute interaction,^40^ each item is rated on a 5-point Likert scale. Higher scores indicate more positive quality in interaction or lack of negative affect or behavior. Scores 1 and 2 describe an area of concern, score 3 an area of some concern, and scores 4 and 5 an area of strength. Two blinded certified coders (including J.L.) rated the data, and 21% of the data were double scored. The coders agreed on 82% of the items on a categorical level. The 5-point scale was used in the analyses, and items were combined into subscales following the general guidelines in the PCERA manual.^38^ Two dyadic scales were combined into 1 global dyadic scale, producing 5 conclusive subscales: (1) maternal positive affect, sensitivity and responsiveness; (2) maternal negative affect and behavior; (3) infant positive affect, communicative and social skills; (4) infant dysregulation and irritability; and (5) dyadic emotional tone, reciprocity and regulation (eTable 1 in Supplement 2). Internal consistency of the subscales (Cronbach α) ranged between .80 and .89. Scale scores represent the means (SDs) of the included items.

### Statistical Analysis

The IPISTOSS sample size was calculated for the main outcome variable of infant cardiorespiratory stability.^34,41^ Data analysis was performed according to intention to treat. Descriptive statistics were used to present baseline variables. Crude and adjusted effects of the dichotomous SSC vs control treatment variable on the PCERA subscales and on accumulated time of skin contact were estimated with multilevel regression analyses, in which children were nested within mothers, thereby controlling for the dependence of data from twins with the same mother. Effect sizes were estimated with Cohen *d*. Adjustments were made for statistically significant imbalances found between groups in terms of primiparity, child sex, and observation setting (home vs clinic). Through bootstrapping 1000 subsamples from the study sample, we estimated the size and statistical significance of mediated effects of SSC vs controls on PCERA subscales via accumulated time of SSC. The mean of the mediated effect across the 1000 bootstrapped subsamples was divided by the SD of the mediated effect across the subsamples. This gave an estimated *z* score of the mediated effect, used to estimate a *P* value of the effect. *P* < .05 was considered statistically significant with a 2-sided hypothesis test. Analyses and illustrations were conducted with IBM SPSS Statistics, version 28 (IBM Corp), and R statistical software, version 4.1.3 (R Core Team) employing the effsize^42^ and beanplot^43^ packages. Data analyses were performed on March 16 and September 18, 2023.

## Results

### Study Participants

**Figure 1.**
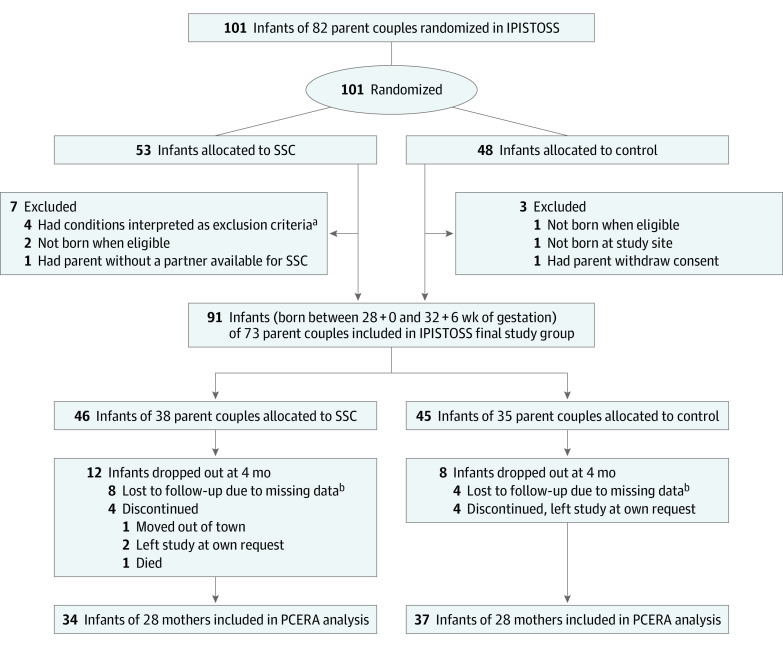
Flowchart of This Secondary Analysis of the Immediate Parent-Infant Skin-to-Skin Study (IPISTOSS) Characteristics of randomization and allocation to the skin-to-skin contact (SSC) intervention or control groups at birth, dropouts at 4-month follow-up, and the final cohort included in the Parent–Child Early Relational Assessment (PCERA) analysis. ^a^The second infant recruited in Sweden was excluded by mistake (data collection was stopped) when the infant needed ventilator care. Three infants in Norway were excluded due to later diagnosis of severe metabolic diseases, interpreted as exclusion criteria. ^b^Missing data were mainly due to challenges for families to come to the clinic for follow-up during the COVID-19 pandemic.

This study included 71 infants (31 twins [44%]) and 56 mothers in the PCERA analysis at 4 months. Infants had a mean (SD) gestational age of 31 weeks 3 (1.3) days and a mean (SD) birthweight of 1535 (408) g; 42 (59%) were boys and 29 (41%) were girls. Mothers had a mean (SD) age of 32 (4.9) years; 32 (57%) were primiparous. A total of 37 infants were allocated to standard care and 34 to SSC with either parent after birth. Baseline characteristics of infants and mothers, including depression symptoms, anxiety symptoms, and parenting stress, were distributed equally between groups, except there were more boys and first-time mothers in the SSC group (Table 1). Figure 1 describes the IPISTOSS study flow and dropouts (20 infants [22%]) at 4-month follow-up. eTable 2 in Supplement 2 compares the group included in the PCERA analysis and the dropout group at 4 months.

**Table 1.** Mother and Infant Characteristics^a^

Characteristic	SSC group (n = 34 infants and 28 mothers)	Control group (n = 37 infants and 28 mothers)
Gestational age, wk + d, mean (SD)	31 + 3 (1.4)	31 + 0 (1.1)
Birthweight, mean (SD), g	1612 (417)	1464 (391)
Birth mode		
Vaginal birth	13 (46)	7 (25)
Cesarean delivery	15 (54)	21 (75)
Apgar score at 5 min, median (IQR)	9 (8-10)	9 (8-10)
Twins	13 (38)	18 (49)
Child sex		
Female	9 (26)	20 (54)
Male	25 (74)	17 (46)
Preeclampsia	8 (29)	10 (36)
Primiparity	20 (71)	12 (43)
Maternal age, mean (SD), y	32 (4.7)	33 (5.1)
Cohabiting parents	27 (96)	27 (96)
University education of mother	17 (61)	22 (79)
Mental health diagnosis mother	5 (19)	3 (11)
Maternal depressive symptoms at 3 mo, mean (SD) (n = 28 and 26)^b^	5.6 (4.6)	6.4 (5.5)
Maternal anxiety symptoms at 3 mo, mean (SD) (n = 28 and 26)^c^	33.9 (10.5)	34.6 (8.6)
Parental stress at 3 mo, mean (SD) (n = 25 and 24)^d^		
Total score	2.8 (0.4)	2.9 (0.3)
Domain		
Sense of competence	2.1 (0.7)	2.0 (0.6)
Role restriction	3.3 (0.9)	3.6 (0.7)
Social isolation	1.9 (0.6)	1.9 (0.5)
Spouse relationship	1.8 (0.7)	1.9 (0.8)
Health	2.8 (0.6)	2.8 (0.5)

Abbreviation: SSC, skin-to-skin contact.

^a^ Unless indicated otherwise, values are presented as No. (%) of participants.

^b^ Measured with the Edinburgh Postnatal Depression Scale (score, 0-30).

^c^ Measured with the Spielberger State-Trait Anxiety Inventory (score, 20-80).

^d^ Measured with the Swedish Parenthood Stress Questionnaire (score 0-5, with 5 indicating highest level of stress).

### Initiation and Duration of SSC During the Intervention and First 8 Days After Birth

For infants in the SSC group, SSC was initiated at a median (IQR) time of 15 (0-62) minutes after birth. During the intervention period of 0 to 6 hours, the median (IQR) SSC duration was 5.25 (4.5-5.5) hours, and fathers provided more SSC than mothers during this period (3.25 [2.25-4.5] vs 0.75 [0-2.5] hours, respectively). Table 2 presents the median SSC duration in the SSC and control groups during the first 6 hours and post intervention (during the first 72 hours and first 8 days) after birth for mothers, fathers, and infants, respectively.

**Table 2.** Duration of SSC During Intervention 0 to 6 Hours After Birth and Accumulated Within First 72 Hours and First 8 Days

SSC duration	Control group (n = 37 infants and 28 parent couples)			SSC group (n = 34 infants and 28 parent couples)		
	Mother	Father	Infant	Mother	Father	Infant
Intervention 0-6 h after birth, median (IQR)	0	0	0	0.75 (0-2.5)	3.25 (2.25-4.5)	5.25 (4.5-5.5)
Accumulated, median (IQR)^a^						
7-72 h after birth	5.25 (3.5-9.5)	3 (0-4.75)	10 (5.25-13.5)	9.5 (5.25-16.25)	8.75 (2.5-11)	17 (10.5-25)
7 h to 8 d after birth	19.5 (15-28.5)	12.5 (8.25-17.5)	36.5 (24.75-44)	29.25 (20-42.75)	23 (10.75-29.75)	51.75 (36.5-70)

Abbreviation: SSC, skin-to-skin contact.

^a^ In the SSC group, data were missing for 2 mothers, fathers, and infants 7 to 72 hours after birth and for 4 mothers, fathers, and infants 7 hours to 8 days after birth.

### Primary Analysis: Immediate SSC and Mother-Infant Interaction at 4 Months

**Figure 2.**
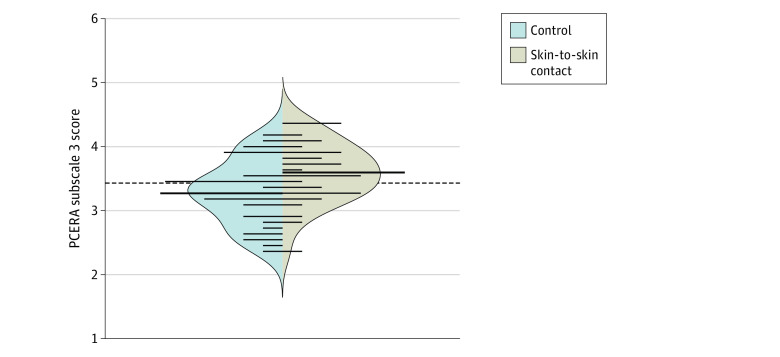
Differences Between the Skin-to-Skin Contact and Control Groups on Parent–Child Early Relational Assessment (PCERA) Subscale 3 (Infant Positive Affect, Communicative and Social Skills) For each group, the beanplot figure displays the mean (bold horizontal lines), the spread of individual observations (small horizontal lines), and the shape of the distributions.

Table 3 presents descriptive statistics and allocation differences for the SSC and control groups on the 5 PCERA subscales. A significant difference in PCERA subscale 3 scores (infant positive affect, communicative and social skills) was observed (Cohen *d* = 0.67 [95% CI, 0.17 to 1.17]; *P* = .01), with higher-quality interaction in the SSC group (Figure 2). After adjustment for primiparity, child sex, and observation setting, this effect remained significant (Cohen *d* = 0.67 [95% CI, 0.15-1.17]; *P* = .02). For PCERA subscale 5 (dyadic emotional tone, reciprocity and regulation), there was no significant difference in the unadjusted analysis (Cohen *d* = 0.56 [95% CI, 0.08 to 1.04]; *P* = .07); however, the difference became significant (Cohen *d* = 0.56 [95% CI, 0.05-1.07]; *P* = .04) after adjustment for observation setting. No other significant differences between the study groups were observed.

**Table 3.** Mother-Infant Interaction at 4 Months for the SSC and Control Groups, by PCERA Subscale

Variable	PCERA subscale, mean (SD)				
	1. Maternal positive affect, sensitivity and responsiveness	2. Maternal negative affect and behavior	3. Infant positive affect, communicative and social skills	4. Infant dysregulation and irritability	5. Dyadic emotional tone, reciprocity and regulation
Control group (n = 37 infants and 28 mothers)	3.24 (0.49)	3.90 (0.52)	3.27 (0.50)	4.20 (0.58)	3.46 (0.52)
SSC group (n = 34 infants and 28 mothers)	3.46 (0.40)	4.03 (0.28)	3.60 (0.47)	4.39 (0.52)	3.71 (0.35)
Cohen *d* (95% CI)	0.50 (−0.02 to 1.02)	0.32 (−0.16 to 0.80)	0.67 (0.17 to 1.17)	0.35 (−0.14 to 0.83)	0.56 (0.08 to 1.04)
*P* value^a^					
Unadjusted	.10	.32	.01	.15	.07
Adjusted					
Primiparity	.10	.36	.008	.11	.05
Child sex	.21	.58	.04	.29	.14
Observation setting	.11	.29	.006	.10	.04
All	.25	.59	.02	.15	.06

Abbreviations: PCERA, Parent–Child Early Relational Assessment; SSC, skin-to-skin contact.

^a^ Unadjusted and adjusted *P* values of the difference between groups.

### Exploratory Analysis: Accumulated SSC Duration Within 8 Days After Birth and Mother-Infant Interaction at 4 Months

After the intervention, infants’ accumulated time of SSC was higher in the SSC group during the first 72 hours, with a median (IQR) of 17 (10.5-25) vs 10 (5.25-13.5) hours in the control group (Cohen *d* = 1.04 [95% CI, 0.53 to 1.56]; *P* < .001). This group’s accumulated time of SSC was also higher during the first 8 days, with a median (IQR) of 51.75 (36.5-70) vs 36.5 (24.75-44) hours (Cohen *d* = 0.92 [95% CI, 0.40 to 1.43]; *P* < .001). Two mediation analyses were performed to test whether the effect of SSC vs the control on PCERA subscale 3 was mediated through the accumulated SSC time within the first 72 hours and 8 days after birth. No significant effects were observed.

## Discussion

This protocol-based secondary analysis of a multicenter randomized clinical trial investigated the effect of immediate SSC with a parent after birth on interaction between very preterm infants and their mother. Our findings support the existence of an early sensitive period for very preterm infants after birth. Infant expressions of positive affect, communication, and social skills were most favorable in the mother-infant dyads allocated to immediate SSC. The dyadic interaction was more optimal in the SSC group after adjustment for observation setting. Maternal contributions to interaction quality did not differ between groups. Although SSC initiated at birth was also associated with longer SSC duration during the first week after birth, no effects of accumulated SSC after the first 6 hours on mother-infant interaction were observed.

Sensitive periods have been defined as opportunities that exist in early environments. These opportunities are biobehavioral experiences between parents and infants that trigger specific neuroendocrine systems influencing gene expression, brain development, and parent-infant attachment.^29,44^ Animal studies have shown how close maternal contact after birth helps organize the physiological systems, stress response, and social orientation of offspring.^45,46^ For human neonates, SSC with their mother after birth has been suggested to provide the expected environment for optimal early development, and the effects of interventions involving parent-infant proximity during this period may be particularly strong on infant brain function.^47^ Mehler et al^33^ found that 60 minutes of mother–preterm infant SSC at birth had beneficial effects on mother-infant interaction at 6 months, with improved infant motor and vocal development. In the same population, reduced long-term expression of stress response genes was found in the SSC group.^48^ That study is, to our knowledge, the only randomized clinical trial to describe effects of SSC at birth on mother-infant interaction in a very preterm population.^33^ However, in our study, infants remained in SSC longer (during the first 6 hours) to further support their physiological transition after birth,^41,49^ and SSC was provided by both mothers and fathers.

In this study, enhanced interaction quality in the SSC group was observed for infant PCERA subscale 3, which describes positive affect and communicative and social skills. Furthermore, after adjustment for observation setting, a between-group difference was observed for dyadic PCERA subscale 5, which describes emotional tone, reciprocity and regulation between mother and infant. Thus, immediate SSC seems to be especially beneficial for interactive behaviors of very preterm infants, and it may also benefit the dyadic aspects of interaction. One possible interpretation is that infants exposed to SSC at birth became more mature social partners, making it easier for their mother to respond to and interact with them. As reported previously, preterm infants have an increased risk of less synchronous interactions due to their immature nervous system and diffuse behavioral cues.^50^ In a meta-analysis,^51^ SSC generally had a positive effect on preterm infant self-regulation skills, such as emotion regulation, which is important for social interaction. The positive development in infant interactive behaviors may contribute to more positive dyadic interaction, because these infants may appear more engaging to their mother.

Interestingly, in our study, fathers provided the most SSC at birth. This finding reflected the clinical situation, with mothers often unavailable for SSC during the first hours after a cesarean birth, for example. The beneficial effect of SSC on interaction was assessed in the mother-infant dyad 4 months later. Our findings showed that the higher-quality mother-infant interaction in the SSC group was driven by the infants’ enhanced social skills. Thus, an important conclusion of the present data might be that from a developmental perspective, time spent in SSC after birth is valuable and may be provided by either parent. This finding highlights the role of fathers in supporting the development of their very preterm infant immediately from birth when the mother is not available,^52^ and it also points to the triadic nature of family relationships.^53^

### Strengths and Limitations

This study had several strengths, including its randomized design, which ensured substantially decreased selection bias. The study was well controlled, since only the place of care differed during the intervention. At the 4-month follow-up, 20 (22%) of the originally randomized infants were lost to the PCERA analysis; however, no differences between analyzed infants and dropouts were found, minimizing the risk for attrition bias in this study. Another strength was the robust observational measurement tool used. The PCERA is well used in studies worldwide and suitable for the preterm population,^54,55^ and it allowed for an objective assessment of mother and infant behaviors. Interrater reliability between the 2 blinded coders was high, as was the internal consistency of the PCERA subscales.

This study had limitations. The sample size was small, due to the main trial being terminated earlier because of benefit of the intervention.^41^ The current exploratory analysis of accumulated SSC was not prespecified, with a chance of a type 2 error given the sample size. We applied the same exclusion criteria for both groups, but 4 infants in the intervention group were excluded due to health conditions; this may have influenced the results either way. Interactions were video recorded both in the clinic and in participant homes; we could not always use the preselected toys because they were not allowed into homes due to COVID-19 restrictions. These limitations may have caused some variability in PCERA scores, so we adjusted for setting in the analysis. Also, dyadic interaction was observed only in a free-play situation, which might limit the generalizability of these findings to other (ie, more stressful) caregiving situations.^56^ The study population is from a high-income setting offering high-quality neonatal care, and the results should be interpreted within this context. Finally, although most time in SSC after birth was spent between fathers and infants, the effect of SSC was evaluated only with the mother-infant dyad. Still, the early exposure to SSC was clearly associated with higher scores of communication skills and positive affect in infants. Future research should investigate the effects of immediate SSC on the father-infant relationship as well as on parent-infant interaction in diverse caregiving settings and situations.

## Conclusions

In this secondary analysis of the IPISTOSS randomized clinical trial, SSC practiced between a parent and a very preterm infant in the immediate postpartum period after birth enhanced child interactive behaviors and positive affect at 4 months of corrected infant age. Skin-to-skin contact may also benefit the dyadic aspects of interaction. These findings support the existence of a sensitive period after very preterm birth, during which close contact between parent and infant may induce a long-term positive effect on the parent-infant relationship. To support infant long-term development, clinical practices should consider the place of care to be in immediate direct SSC with a parent after birth, alongside other necessary nursing and medical care.

## Notes

Supplement 1.
Trial Protocol

Supplement 2. **eTable 1.** Parent–Child Early Relational Assessment (PCERA) Items Assessed in a Free-Play Situation at 4 Months of Corrected Infant Age **eTable 2.** Comparison of Mother and Infant Characteristics of the Group From the IPISTOSS Study Included in the Parent–Child Early Relational Assessment Analysis at 4 Months of Corrected Infant Age (PCERA Group) and the Group That Did Not Participate in the 4-Month Follow-Up (Dropout Group)

Supplement 3.
Data Sharing Statement
