# Supplementary material for: Skin-to-Skin Contact at Birth for Very Preterm Infants and Mother-Infant Interaction Quality at 4 Months: A Secondary Analysis of the IPISTOSS Randomized Clinical Trial
Source: JAMA Netw Open. 2023 Nov 30;6(11):e2344469. doi: 10.1001/jamanetworkopen.2023.44469 (PMC10690460; doi:10.1001/jamanetworkopen.2023.44469)
Supplement: Supplement 1. — Trial Protocol [file jamanetwopen-e2344469-s001.pdf]

IPISTOSS – Immediate Parent-Infant Skin-to-skin Study

Management: Skin-to-Skin Contact with a parent during the first hours after birth for infants born between 28+0 and 32+6 weeks gestation.

Sponsor: Björn Westrup

Coordinating investigator: Wibke Jonas

Principal investigators: Agnes Linnér and Siren Rettedal

Contents

1 Summary **4**

2 ABBREVIATIONS **5**

3 AdministrativE information **6**

4 BACKGROUND **6**

4.1 Condition 6
4.2 Intervention 7
4.3 Aim of the study 7

5 HYPOTHESES **8**

5.1 Primary hypothesis 8
5.2 Secondary hypotheses 8

6 ENDPOINTS **9**

6.1 Primary endpoints 9
6.2 Secondary endpoints 9

7 METHODS **10**

7.1 Method overview 10
7.2 Assessment and procedures 11
7.3 Flow chart 13
7.4 Trial completion 13

8 TRIAL SUBJECTS **13**

8.1 Inclusion criteria 13
8.2 Exclusion criteria 13
8.3 Criteria for interruption and cessation 14
8.4 Recruitment register 14
8.5 Description of treatment 14
8.6 Determination of allocation 14
8.7 Blinding and breaking code 14
8.8 Concomitant study interventions and medications 15
8.9 Compliance 15
8.10 Treatment after the study 15

9 ASSESSMENT OF SAFETY AND EFFECTIVENESS **15**

9.1 Assessment of clinical effectiveness 15
9.2 Assessment of clinical safety 16
9.3 Assessment of results- effectiveness parameters 16
9.4 Assessment of analysis results – safety parameters 17

10 MANAGEMENT OF ADVERSE EVENTS **17**

10.1 Definitions 17
10.1.1 Adverse event (AE) 17
10.1.2 Serious adverse event (SAE) 17
10.2 Evaluation of adverse events 17
10.2.1 Evaluation of severity 17
10.2.2 Evaluation of association to intervention 17
10.3 Methods to discover adverse events 18
10.4 Reporting of adverse events 18

10.4.1 Reporting of adverse events 18
10.4.2 Reporting of serious adverse events 18
10.5 Follow-up of adverse events 18

11 STATISTICS AND DATA MANAGEMENT 19
11.1 Data management 19
11.2 Statistiscal analysis 19
11.3 Determination of sample size 19

12 ACCESS TO SOURCE DATA 20

13 QUALITY CONTROL 20
13.1 Source data 20
13.2 Monitoring 20

14 ETHICS 20
14.1 Ethics review board 20
14.2 Ethical conduct of the study 20
14.3 Risk benefit evaluation 21
14.4 Trial subject information and consent 22

15 DATA MANAGEMENT AND ARCHIVING 22
15.1 Case Report Forms 22
15.2 Archiving 22

16 FinanCING AND INSURANCE 22

17 PUBLICATION OF RESULTS 22

18 OTHER 22
18.1 Additions to the protocol 22
18.2 Research staff information 23

19 REFERENCES 24

20 SIGNATURES 25

1 Summary

PROTOCOL IDENTITY AND AIM

Protocol title: IPISTOSS

Aim of study: To determine if skin-to-skin contact results in a better physiological stabilization, altered epigenetic profile and better long-term neurodevelopmental outcome.

INTERVENTION

Skin-to-skin contact with a parent from birth

METHODS

Study design: randomized controlled trial.

Duration: Continuous skin-to-skin contact with any parent during the first six hours of life, and as much as possible the first three days.

Primary hypothesis: Does cardiorespiratory stabilization improve?

Effectiveness outcomes: all other outcomes.

Safety parameters: respiration, thermal control, infections.

STUDY POPULATION

Study subjects: Infants born with gestation between 28+0-and 32+6 weeks at Karolinska University Hospital (Danderyd, Huddinge and Solna) in Stockholm, or at Stavanger University Hospital in Stavanger

Number: 150

TIME FRAME

First subject enrolled: 20170501

Last subject enrolled: 20201031

Last subject completed follow-up: 20230131

2 ABBREVIATIONS

Abbreviation	Explanation
AE	Adverse Event
APIB	Assessment of Preterm Infants’ Behavior
SAE	Serious Adverse Event
ASQ	Ages and Stages Questionnaire
BSES	Breastfeeding Self Efficacy Scale
CMM	Centrum för Molekylärmedicin (Centre for Molecular Medicine)
CPAP	Continuous Positive Airway Pressure
CRF	Case Report Form
EEG	Electroencephalography
EPDS	Edinburgh Postnatal Depression Scale
HRV	Heart Rate Variability
IBFAT	Infant Breastfeeding Assessment Tool
IBS	Index of Breastfeeding Status
IBQ	Infant Behaviour Questionnaire
KMC	Kangaroo Mother Care
KTA	Karolinska Trial Alliance
MCHAT	Modified Checklist for Autism in Toddlers
PCERA	Parent-Child Early Relational Assessment Scale
PKU	Phenylketonuria (and term for metabolic screen)
RCT	Randomized Controlled Trial
SCRIP	Stability of the CardioRespiratory system In the Preterm
SOP	Standard Operating Procedure
SPSQ	Swedish Parenthood Stress Questionnaire
SSC	Skin-to-Skin Contact
STAI	The Spielberg State-Trait Anxiety Inventory
SUPC	Sudden Unexpected Postnatal Collapse
WHO	World Health Organization

3 ADMINISTRATIVE INFORMATION

Sponsor: Björn Westrup, MD PhD, PO Sjuka Nyfödda Barn/Institutionen för Kvinnors och Barns Hälsa
Prinicpal investigator in Stockholm (Danderyd and Huddinge): Agnes Linnér, MD and PhD student, PO Sjuka Nyfödda Barn/Institutionen för Kvinnors och Barns Hälsa
Prinicpal investigator in Stavanger: Siren Rettedal, MD PhD, Stavanger Universitetssykehus, Norway
Prinicpal and coordinating investigator in Stockholm: Wibke Jonas, PhD, associate professor, Institutionen för Kvinnors och Barns Hälsa
Co-investigator Stockholm och Stavanger: Nils Bergman, MD, Cape Town
Co-investigator Stockholm: Stina Klemming, MD, PO Sjuka Nyfödda Barn
Co-investigator Stockholm: Siri Lilliesköld, RN, PhD student, PO Sjuka Nyfödda Barn
Co-investigator Stavanger: Hanne Pike, MD, PhD student, Stavanger Universitetssykehus, Norway
Co-investigator Stavanger: Karoline Lode, MD, Stavanger Universitetssykehus, Norway

Oversight of secondary outcomes:
Physiological outcomes: Nils Bergman, MD, Cape Town, South Africa.
Parent child interaction, parental health, breastfeeding and stress biology: Wibke Jonas, PhD, associate professor, Institutionen för Kvinnors och Barns Hälsa
Epigenetics: Malin Almgren, PhD, assoc professor, CMM, Karolinska Universitetssjukhuset
Microbiota: Lars Engstrand, professor, Institutionen för Mikrobiologi, Tumör- och Cellbiologi, Mattias Larsson, MD, PhD, associate professor, Institutionen för Folkhälsovetenskap, Linus Olsson, PhD, post doc, Institutionen för Folkhälsovetenskap
Brain imaging /MRI: Ulrika Ådén, MD Professor, PO Sjuka Nyfödda Barn/Institutionen för Kvinnors och Barns Hälsa Nelly Padilla, MD PhD, Institutionen för Kvinnors och Barns Hälsa
Stress reactivity/salivary cortisol: Evalotte Mörelius, PhD, specialist nurse children and adolescents. Professor of Nursing, Children and Young People vid Edith Cowan University och Perth Children`s Hospital. E-postadress: evalotte.morelius@liu.se, e.morelius@ecu.edu.au
Psychomotor long term outcomes: Ulrika Ådén, MD Professor, PO Sjuka Nyfödda Barn/Institutionen för Kvinnors and Barns Hälsa, Wibke Jonas, PhD, associate professor, Institutionen för Kvinnors och Barns Hälsa

Research nurse in Stockholm: Kerstin Andersson, RN, Institutionen för Kvinnors och Barns Hälsa
Research nurse in Stavanger: Kirsten Engevik, RN, Stavanger Universitetssykehus
Statistician: Jan Kowalski, EDC Scandinavia AB
Monitor: Karolinska Trial Alliance in Stockholm. Awaiting decision on monitor in Stavanger.

See Appendix 1 for complete contact details.

4 BACKGROUND

4.1 Condition

Infants born very preterm are cared for in the neonatal ward in their first weeks and months of life. Being born too soon means that those organs that would have developed in the uterus must instead do so in the hospital environment. This requires many aspects, including support for breathing, enteral and parenteral nutrition, humidity and warmth, monitoring, gentle supportive care and proximity to parents. The first few hours of life are especially important, during which transition to extrauterine life takes place, the infant's breathing and circulation changes from reliance on the lungs rather than the placenta. Events taking place in these first few days and weeks do impact future health and family relationships. The consequences of prematurity can result in symptoms from many organs in the short and long term, but above all long-term brain development can be adversely affected, with psychomotor impairments (1).

Current practice is to care for premature infants in incubators. The incubator provides needed humidity and warmth, but it also results in abrupt separation from mother. Empirically, it is observed that premature infants and

their parents benefit from intermittent skin-to-skin contact during the stay in the neonatal wards. For full term newborns it is well established that skin-to-skin contact is beneficial (2). For preterms, initiation of skin-to-skin contact and the number of hours per day it is provided depends on infants gestational age and medical condition as well as the circumstances of the ward and the parents.

In Swedish the term ”*hud-mot-hudkontakt*” is the direct translation of ”skin-to-skin contact” (SSC). The term ”Kangaroo Mother Care” (KMC) is often used as a synonym, however the definition of KMC is a broader concept including support for breastfeeding and early discharge from hospital.

4.2 Intervention

Intervention:
The intervention in this study is the place of care, the exact same care is given but in SSC with a parent or surrogate instead of resuscitaire or incubator. This will be continuous for the first six hours of life, and as much as possible during the first 72 hours.

Previous studies:
In a recently updated Cochrane-review a 40% lower mortality is reported at term equivalent age in infants born below 2kg cared for in SSC/KMC compared to conventional care (3,2% vs 5,3%; RR 0,60, 95% CI 0,39-0,92; 8 studies, 1239 infants), a shorter hospital stay, and higher prevalence duration and degree of breastfeeding (3). In almost all these studies, KMC was initiated when the infant was stable. The median age of initiation was 3,2 to 24,5 days, only in one study was KMC initiated around 10 hours (4). The implication is that two thirds of deaths among preterm infants would have occurred before the time any infant could have been assessed as stable enough for KMC (5).

Two studies from middle income countries report on SSC and physiological stabilization. One RCT from South Africa enrolled infants with birthweight 1200-2199 g, in the first 6 hours of life: those in SSC had 100% stability scores compared to 46% for those in incubators. Those in incubators also had significantly lower temperatures (6). In a similar study from Vietnam infants with birthweight between 1500-2500 g in the SSC group had significantly improved scores for transition to extrauterine life. Further, they required less respiratory support, intravenous fluids and antibiotics during hospital stay (7).

This current study is ongoing since May 2017 in Stavanger, Norway, and since November 2018 at Karolinska University Hospital in Huddinge, Stockholm, Sweden.

Risks of the intervention.
The main risk factor for immature and unstable infants during SSC is obstructive apnoea, from a position failing to ensure patent airway. To avoid this all infants will be positioned correctly, and during transfer or transport we will ensure fixation on parent or surrogate with a garment. All infants will be monitored ongoing with pulse oximetry. Preterm and low birth weight infants are susceptible to hypothermia if the environment is too cold. They should therefore be covered with a blanket when receiving SSC. A consequence of this is that staff may have difficulty to visually assess the condition of the infant as is possible in open or closed incubator. Therefore, staff will make regular observation of the infant in the SSC position, as they would for incubator infants. There is further a risk that the interpretation of the infant’s condition is misinterpreted, as unstable infants were not previously in SSC.

Benefits of the intervention.
Benefits of the study could be that the early SSC enhances the physiological transition from intrauterine to extrauterine life and protects against some of the medical problems associated with preterm birth. Another benefit could be a reduced time of separation allowing parents greater opportunity to bond to their infants.

4.3 Aim of the study

The context for this study is the global health situation for preterm and low birth weight infants. Most of these are born in developing countries and have a high mortality rate. The high technology care provided in developed countries is seen as a standard of care, even though it can seldom be provided due to lack of resources. Healthy full-term infants are often traditionally cared for with the mother from the beginning, and often in SSC. Should the infant be born preterm, or by caesarean section, or be sick in any way it is admitted to neonatal wards that are generally already full of sick infants. Mothers have limited access to their infants and are usually only allowed in at feeding times. The numbers of infants in relation to staff and space contributes to morbidity and mortality that could have been prevented with relatively simple measures (8). In many parts of the world there are KMC wards established for more mature and stable infants for ongoing SSC as support for breastfeeding and weight gain while awaiting discharge. WHO recommends that all stable low birth weight infants to be kept in SSC (9). A study is currently underway in low- and middle-income countries looking at survival rates from immediate SSC (Immediate KMC Study) under the sponsorship of the WHO.

In Sweden intermittent SSC is used in neonatal intensive care units and small infants are seen to do well. There is however very little research on immediate SSC on infants born below 33 weeks gestational age. We wish to study what happens if an earlier start of this simple and inexpensive intervention is introduced. The study population will be infants born between gestational age 28+0 to 32+6 weeks, approximating 1-2 kilograms in weight. The aim is to conduct a study in a high-income country context to elucidate more advanced effects and underlying mechanisms as well as the safety of the method in a manner that is not possible in low income countries with scarce resources. This study therefore complements the abovementioned WHO study on survival.

5 HYPOTHESES

5.1 Primary hypothesis

Does skin-to-skin contact during the first six hours lead to improved cardiorespiratory stabilization?

5.2 Secondary hypotheses

The secondary hypotheses will examine whether SSC leads to differences in:

- Respiratory function (need for respiratory support, surfactant, time on ventilator, CPAP and extra oxygen use)
- Heart Rate Variability (HRV)
- Thermal regulation
- Infections
- Breastfeeding, nutrition and growth
- Epigenetic profile after birth and later in development
- Microbiome
- Brain maturation, EEG
- Structural maturation of brain on MRI
- Attachment and interaction between parents and infants

- Brain functions in areas reflecting sensitive parenting (fMRI)
- Parents' experiences and mental health
- Capacity in infants and mothers to regulate stress and interaction
- Psychomotor development (neurology, motor function, cognition, behaviour, language)

6 ENDPOINTS

6.1 Primary endpoints

Cardiorespiratory stability is measured with the SCRIP score (Stability of the CardioRespiratory system In the Preterm), a scale that summates the respiratory (respiratory rate, oxygen saturation, need for respiratory support) and circulatory (pulse) variables in the infant (7). Observations are made during a five-minute period every 15 or 30 minutes during the first six hours, according to prepared template. For each five-minute period the recorded parameters are used to calculate a summary score of physiological stability.

6.2 Secondary endpoints

The time points below are the *corrected age* of the infant, calculated from expected date of full-term birth (not the chronological age calculated from actual date of premature birth). For some secondary outcomes only a subsample of infants will be studied.

Respiratory function: Need for surfactant, number of days in ventilator, number of days in CPAP, number of days requiring oxygen.

Heart rate variability: Postpartum, after 48-72 hours, at discharge and at 3-4 months.

Thermal regulation: Axillary temperature is measured continuously, alternatively every hour on infant, and if in SSC also on parent.

Infections: Proportion of infants that experience sepsis during admission in the neonatal ward according to case record, and total number of days treated with antibiotics.

Breastfeeding, nutrition, and growth: Age at first feed, age at first passage of meconium, time to full enteral feeding, time to weaning from feeding tube, lowest weight, time to return to birth weight, weight gain in grams per day during admission, and weight at discharge as well as term age as per case record. Nutrition and breastfeeding status will be completed weekly according to the Index of Breastfeeding Status (IBS) questionnaire during hospital stay, at discharge, at term age, at 3-4 months of age and at 12 months. The Breastfeeding Self-Efficacy Questionnaire (BSES) will be completed at 7 days, 3-4 months, and the Infant Breastfeeding Assessment Tool (IBFAT) each week during admission, at discharge, at term age and at 3-4 months.

Epigenetics: Whole genome analysis as well as locis specific methylation of stress related genes for Oxytocin receptor (OXTR), glucocortcoid receptor (NR3C1), Arginine-vasopressin receptor (AVP), Proopiomelanocortin (POMC), Glutamate decarboxylase-1 (GAD1), Glutamate receptor (GRM1), Brain-Derived Neurotrophic Factor-(BDNF), serotonin transporter (SLC6A4 och SERT), serotonin receptor (5H2A) och serotonin degradation enzyme (MAOA) in cord blood postpartum, in buccal swabs postpartum and at 6 hours, whole blood from opportune time during first 6 hours, whole blood and buccal swabs at 48-72 hours, at 3-4 months and at 24 months of age.

Microbiota: Infant colonization by microbiome (bacteria) is identified by DNA analysis from fecal samples at 0-6 hours, 72 hours, 3-4 months, 12 och 24 months, and compared to the parents. Vaginal, rectal, and skin samples from mother and partner are collected at 0-6 hours.

Functional maturation of brain: EEG-activity at 4-10 days of age and at term age.

Structural maturation of brain: MR brain at term age.

Attachment: Attachment as assessed by video filming at 3-4 months of age in conjunction to the ”Still Face-test”, as well as interaction as interpreted by the Parent-Child Early Relational Assessment Scale (PCERA), which will also be done at 12 and 24 months of age.

Brain function: Functional magnetic resonance imaging (MRI) to demonstrate activation patterns in specific areas in maternal brain when she studies pictures of her own and other children in different emotional states, when her infant is 3-4 months old.

Parental mental health: Experience of care as assessed by interview at discharge. Further according to Edinburgh Postnatal Depression Scale (EPDS) at 7 days, 3-4 months and 12 months of infant age, the Spielberg State-Trait Anxiety Inventory (STAI) at 7 days, 3-4 months and 12 months of infant age and the Swedish Parenthood Stress Questionnaire (SPSQ) at term age, 3-4 months and 12 months of infant age.

Stress reactivity: According to salivary cortisol levels in infant and mother on the morning and evening near day of hospital discharge, and in the same day before and after nappy change (stressor), at 3-4 months of age, and the same day as the Still Face-test (stressor), and at 12 months of age.

Infant psychomotor development: as recorded in hospital case record at term age, and again at 3-4 months, 12 months and 24 month of age, further according to Assessment of Preterm Infant Behaviour (APIB) at term age, according to Infant Behaviour Questionnaire (IBQ) and Ages and Stages Questionnaire (ASQ) at 3-4 months (ASQ 3-8) and 12 months (ASQ 9-14)as well as developmental assessment according to Bayley Scales of Infant and Toddler Development 3rd Edition, and Modified Checklist for Autism in Toddlers (MCHAT) at 24 months age.

7 METHODS

7.1 Method overview

This is a prospective multicenter randomized controlled trial. Screening for study subjects takes place among women with threatening premature birth in the antenatal and labor ward, with randomization taking place antenatally. The study population is 150 infants (75 infants in control and in intervention) born between gestational age 28+0 and 32+6 weeks at Karolinska University Hospital labour wards in Danderyd and Huddinge in Stockholm and at Stavanger Universityhospital in Norway. The intervention is skin-to-skin contact with either parent or with a surrogate chosen by them, the SSC should be uninterrupted during the first six hours of life, and as much as possible in the first 72 hours. Data will be collected during this time, and then during the period of admission, and continued until the infant reaches 24 months corrected age (described further below). Certain selected outcomes will be studied in subsamples of the 150 subjects, and for practical reasons some at only one site. Some families studied may later be transferred elsewhere in the country, data for these will continue to be collected as far as possible. There may also be certain exclusions due to language and interpreter problems that may influence data. Discussion on sample sizes for subsamples is ongoing.

7.2 Assessment and procedures

Note that in the following section the corrected age is used, that is to say time from the expected date of full-term birth, and not the chronological age calculated from the actual premature date of birth.

Antenatal:

- Information about the study.
- Gathering of consent.
- Gathering of background information (parent's education, information about family, address, smoking, earlier illnesses and pregnancy related problems from the mother, antenatal corticosteroids).
- Randomization when birth is inevitable and imminent, at latest one hour before birth.

As soon as possible after birth:

- Cord blood and buccal swab is taken from the infant for epigenetic testing.
- Fecal sample and skin swab taken from the infant for analysis of microbiota
- Maternal samples from rectum, vagina and skin for microbiota analysis.
- Father or partner swabbed for microbiota analysis.
- HRV

From birth through the first six hours of life:

- Intervention or conventional care. Intervention is continuous skin-to-skin contact.
- Physiological parameters collected by direct observation of infant and by pulse oximetry.
- Axillary temperature measured continuously and recorded every hour for infant, and for SSC group also on the parent.
- At any stage during the first 6 hours, when normal routine whole blood collection is undertaken, an additional sample is taken for epigenetic analysis.

At 6 hours of age

- Buccal swab is taken from the infant for epigenetic analysis.

From 6-72 hours of age

- Intervention continued, or conventional care recorded. The intervention is intermittent SSC and striving to achieve continuous. Time in SSC and time in any other place is carefully recorded.
- Clinical information is collected from observations, or from case record review.
- Information on first feeding, on type of feeds given, and first passage of meconium is recorded.

At 48-72 hours (in conjunction with the PKU-test):

- Buccal swab and whole blood sample for epigenetic analysis.
- Fecal sample, and nasal and skin swab taken for microbiota analysis.
- HRV

Age 4-10 days:

- EEG

Age 7 days:

- Breastfeeding questionnaire BSES completed.
- Questionnaires EPDS och STAI completed.

Each week during admission:

- Nutritional status including questionnaires IBS and IBFAT completed.

At discharge:

- Information on need for respiratory support collected from hospital case record.
- Information on infections collected from hospital case record.
- Information on growth collected from hospital case record.
- Skin-to-skin contact time and care in other place during hospital stay collected.
- Lowest weight, current weight and weight gain per day collected from hospital case record.
- Nutritional status including questionnaires IBS och IBFAT complete for hospital stay.
- HRV
- Parents are interviewed about their experience of care, including benefits and challenges with care in skin-to-skin contact.
- Salivary cortisol measured on mother and on infant in the morning and the evening, as well as before and after a nappy change.

At follow-up visit when reached term age:

- HRV
- Psychomotor developmental assessment according to standard hospital follow-up program.
- Observation according to APIB.
- The questionnaires EPDS, STAI and SPSQ completed.
- The questionnaires IBS and IBFAT completed.

At another visit when term age:

- EEG
- Observation according to APIB (if not done at the routine visit)

At another visit when term age:

- Structural maturation of brain according to MRI.

At follow-up visit 3-4 months corrected age:

- Psychomotor developmental assessment according to standard hospital follow-up programme.
- Buccal swab and whole blood collected for epigenetic analysis.
- Fecal sample and skin swab taken from the child for microbiota analysis.
- Questionnaires IBS, BSES and IBFAT completed.
- Questionnaires EPDS, STAI and SPSQ completed.
- Questionnaires ASQ and IBQ completed.

At another visit 3-4 months corrected age:

- Assessment of attachment and interaction between parent and infant done through the ”Still Face”-test.
- Filming and scoring of Parent-Child Early Relational Assessment Scale (PCERA).
- Salivary cortisol is measured morning and evening as well as before and after the ”Still Face”-test.
- HRV measured before, during and after the ”Still Face”-test.

At another visit 3-4 months corrected age:

- Functional MRI of brain of mother.

At routine follow-up visit 12 months corrected age:

- Psychomotor developmental assessment according to standard hospital follow-up program.
- Filming and scoring of Parent-Child Early Relational Assessment Scale (PCERA).
- Fecal sample and skin swab taken from the child for microbiota analysis.
- Salivary cortisol measured on infant and mother.
- Questionnaire IBS completed.
- Questionnaires ASQ and IBQ completed.
- Questionnaires EPDS, STAI and SPSQ completed.

At follow-up visit when 24 months corrected age:

- Psychomotor developmental assessment according to standard hospital follow-up program.
- Filming and scoring of Parent-Child Early Relational Assessment Scale (PCERA).
- MCHAT completed.
- Buccal swab and whole blood collected for epigenetic analysis.
- Fecal sample and skin swab taken from the child for microbiota analysis.

At another visit when 24 months corrected age:

- Developmental assessment according to the Bayley Scales of Infant and Toddler Development

7.3 Flow chart

Attached, see Appendix 2

7.4 Trial completion

The study will be complete when the last enrolled subject comes for follow-up visit at 24 months corrected age, approximately 2 years and 3 months after last enrollment.

8 TRIAL SUBJECTS

8.1 Inclusion criteria

- Birth at gestational age between 28+0 and 32+6 weeks.
- Born in labor ward at study site.
- Consent from prospective parents, with interpreter if required.
- Parent or surrogate available to commence skin-to-skin contact in the first hour of life.

8.2 Exclusion criteria

- Born outside hospital
- Triplets or higher multiples
- Known congenital malformation requiring surgical management.
- Known congenital infection.
- Any other circumstance precluding subject from enrollment according to responsible clinician.

8.3 Criteria for interruption and cessation

The intervention will sometimes not be able to be initiated immediately and will at times require to be temporarily halted for events and procedures that cannot be done safely in SSC. This does not imply withdrawal from the study, as such situations are expected. Prospective parents will have consented to study participation with information about the importance of adhering to allocated place of care in the first six hours of life. Clinical staff will support such adherence. If parents demand otherwise, this may be accepted after individual assessment by the clinically responsible neonatologist as to safety of infant. Actual time in allocated place of care, as well as time in other place of care will be documented. Criteria for temporary interruption of SSC, with intention to continue straight after, will be:

- If the infant requires intubation
- If the infant requires umbilical catheters
- If infant despite adequate measures has hypothermia
- If infant despite support has repeated severe apneoa and bradycardia
- If infant requires any other intervention and this cannot be done safely in SSC, as assessed by ward staff and responsible neonatologist.

Should the parents of any study subject wish to withdraw from the study this will be accepted. Data collected up to that point will be analyzed unless the parents expressly wish otherwise. Clinical staff may also in consultation with research staff choose to terminate a subject's participation under particular circumstances.

8.4 Recruitment register

All women admitted for threatening premature labor will be logged on a screening list and allocated a screening number. All reasons for non-inclusion in the study will be documented on this list.

TREATMENT

8.5 Description of treatment

Not applicable

8.6 Determination of allocation

Randomization will be done by electronic means, close to the actual birth time but at earliest 6 hours before birth and latest one hour after birth.

8.7 Blinding and breaking code

The intervention is such that blinding of allocation is not possible.

8.8 Concomitant study interventions and medications

Participation in other intervention studies will not be permitted unless these are observational studies. There are no pharmacological contraindications to perticpation obs check participation?

8.9 Compliance

Information about the study is given to the parents antenatally as part of obtaining informed consent. At that time the importance of maintaining place of care according to the randomization will be emphasized. Compliance to study protocol and achieving the maximum dose of intervention and control care will also require training and instructions to all ward staff. All staff involved in care will receive practical training on all aspects of the intervention, and recommendations on where what procedures can be done. Time in skin-to-skin, time sent elsewhere and reasons for any transfers will be documented.

8.10 Treatment after the study

After the last data collection at 24 months corrected age there will be no further follow-up of study subjects or their parents.

9 ASSESSMENT OF SAFETY AND EFFECTIVENESS

9.1 Assessment of clinical effectiveness

SCRIP-score: Respiratory rate, heart rate and oxygen saturation are measured and scored in a table, need for respiratory support is part of the table. The average of total scores is analyzed.

HRV: Heart rate variability is measured for an appropriate interval, possibly using the ”Faros 360” and analyzed according to Porges method by the Nurture Science Programme, Columbia University, NY. Average and median values are analyzed.

Respiration: Need for surfactant, number of days in ventilator, number of days in CPAP, number of days requiring supplemental oxygen collected from hospital case record. Proportions and median values will be analyzed.

Temperature: Axillary temperatures will be recorded. Average and median values are analyzed.

Infection: Proportion of infants experiencing sepsis during admission collected from hospital case record, as well as days receiving antibiotics. Average and median values are analyzed.

Breastfeeding and nutrition: Age at first feed, age at first passage of meconium, time to full enteral nutrition, lowest weight, time to removal of feeding tube, time to regained birth weight, weight gain in grams per day during hospital stay, weight at discharge and at term age is collected from the hospital case record. Breastfeeding status is determined from Index of Breastfeeding Status, Breastfeeding Self-Efficacy Questionnaire and Infant Breastfeeding Assessment Tool (IBFAT). A PhD student will be responsible for extracting data from case record (Take Care and Clinisoft). A research nurse will distribute questionnaires and parents will complete them during admission or at home prior to follow-up visits. Proportions are analyzed.

Epigenetics: Whole genome analysis as well as locus specific methylation of stress related genes for Oxytocin receptor (OXTR), glucocortcoid receptor (NR3C1), Arginine-vasopressin receptor (AVP), Proopiomelanocortin (POMC), Glutamate decarboxylase-1 (GAD1), Glutamate receptor (GRM1), Brain-Derived Neurotrophic Factor- (BDNF), serotonin transporter (SLC6A4 och SERT), serotonin receptor (5H2A) och serotonin degradation enzyme

(MAOA) i cord blood postpartum, in buccal swabs postpartum. Midwife or nurse in labour ward collect cord blood concomitant with routine collection. Neonatal nurse collects blood samples at the same time as required clinically, as well as buccal swab during hospital stay or at follow-up visit. Samples are transported to the Unit for ”Medicinsk Epigenetik, Centrum för Molekylärmedicin”, KI. Analysis is undertaken by responsible researcher.

Microbiota: Infant microbiome colonization is identified with DNA analysis from fecal samples and skin swabs, and compared to that of their parents. Samples from infant are collected by ward nurse. Tests from parents are collected by nurse, or by the parents themselves. Samples are sent to the Center for Translational Microbiome Research (Molekylär- och Tumörbiologiskt Centrum; MTC).

Brain function maturation: EEG is measured with 8 leads using existing apparatus in clinical use. The results will be interpreted by the Nurture Science Programme, Columbia University, NY.

Structural maturation of brain: MR brain will be done on a subsample at term age. Results will be interpreted by radiologists.

Attachment: attachment will be assessed by psychologist on films made during the ”Still Face-test” as well as the Parent-Child Early Relational Assessment Scale (PCERA). Filming is done by research staff. A psychologist or other appropriately trained staff at Linköping University or Karolinska Institutet will interpret the films.

Brain function: Functional magnetic resonance imaging (fMRI) will examine activation patterns in specific areas on mother’s brain while she looks at pictures of her own and other infants in different moods. Parents will be invited to radiology department and the examination conducted by radiologists who will assess whether there are any structural differences, with the hypothesis that functional and structural connectivity in parenting behavior specific areas (e.g. prefrontal cortex and amygdala) are related to time in SSC.

Parental mental health: Experience of care will be assessed in interviews at discharge. Questionnaires Edinburgh Postnatal Depression Scale (EPDS), The Spielberg State-Trait Anxiety Inventory (STAI) and the Swedish Parenthood Stress Questionnaire (SPSQ) are administered. A research nurse distributes the questionnaires and parents complete them during hospital stay or at home before follow-up visit. Proportions are analyzed.

Stress reactivity: Salivary cortisol levels measured in infant and mother. A clinical nurse takes the saliva sample during hospital stay or at follow-up. Samples not taken at visit (planned morning and night samples) are collected by parents and sent. Samples are collected at CMM and will later be sent to the University Hospital in Linköping for analysis by immunoassay. Co-variability in mother-infant tests will be analyzed.

Infant psychomotor development: Assessed by APIB; further as described in hospital case record at follow-up visit according to Infant Behaviour Questionnaire (IBQ) and Ages and Stages Questionnaire (ASQ), also developmental assessment according to Bayley Scales of Infant and Toddler Development 3rd Edition and Modified Checklist for Autism in Toddlers (MCHAT). PhD student will collect data from hospital case record. Parents complete questionnaires before follow-up visits. A psychologist will document developmental assessment in case record, a PhD student will collect this. Averages and proportions are analyzed.

9.2 Assessment of clinical safety

Study sponsor will be informed on unexpected and adverse events, their nature, their frequency, and number of study subjects, collated monthly. Serious adverse events (SAE) shall be reported to sponsor within 24 hours. Evaluation is done as described in section 10 (below), only respiration, temperature and infection parameters will be assessed as to clinical safety. Any SAE shall be reported within one week to the Data Safety Monitoring Board (DSMB), who will also receive outcome reports every 6 months to detect any possible large or clinically important difference in the study groups.

9.3 Assessment of results- effectiveness parameters

Blood samples, buccal swabs, salivary samples, fecal samples, and skin swabs are taken by clinical staff and stored in the neonatal unit until transported to the CMM/KI (epigenetics), Linköping University Hospital, The Center for Translational Microbiome Research/MTC (microbiota). This includes also samples taken in Stavanger. All samples are encompassed by existing biobank regulations in each country, and authorization has been given. Clinical data is collected from hospital case record. Information on attachment, parental health and part of psychomotor development is collected from case record, other part from questionnaires and films. MR data is analyzed by neuroradiology specialists at Karolinska University Hospital and Stavanger University Hospital. Many outcomes are of a nature that reference values and detection limits do not apply, rather evaluation is by association studies and exploratory analysis.

9.4 Assessment of analysis results – safety parameters

For all outcomes safety margins and intervals do not apply. Should any result be abnormal or indicate need for investigation or management, subject will be promptly referred.

10 MANAGEMENT OF ADVERSE EVENTS

10.1 Definitions

10.1.1 Adverse event (AE)

Every adverse clinical event or deterioration of existing medical condition, regardless of any connection to study intervention.

10.1.2 Serious adverse event (SAE)

Every unintended medical event that:

- Results in death
- Is life-threatening
- Leads to a prolonged hospital stay
- Results in permanent or temporary disability
- Leads to injury or deformity
- Is serious for any other reason

10.2 Evaluation of adverse events

10.2.1 Evaluation of severity

Every adverse event shall be classified by the researcher as mild moderate or severe.

Mild: The event does not impact the subjects´ normal life.

Moderate: The event causes deterioration in function but does not impact health. The event gives discomfort and or impediment.

Severe: The event leads to deterioration of function or work capacity or constitutes a health risk for the subject.

10.2.2 Evaluation of association to intervention

Likely: Clinical event, including abnormal laboratory results that occur within a plausible time after the intervention or study product. Unlikely that the event can be attributed to underlying medical conditions or pharmaceuticals.

Possible: Clinical event, including abnormal laboratory tests, that occurs within a reasonable time following the intervention or study product. The event can be attributed to underlying medical condition or to other drugs.

Unlikely: Clinical event, including abnormal laboratory tests, that could potentially be related to the intervention or study product. The event is unlikely to be related to the intervention or study product and can be explained by other drugs or by the underlying medical condition.

Not possible to classify: The event cannot be classified due to lack of information, or the event is not verified.

10.3 Methods to discover adverse events

<u>Adverse events are:</u>

- Death
- Cardiorespiratory deterioration with need for increased intensive care, for example apnea obs vi har ett stavfel högre upp with need for ventilation, circulatory collapse with need for bolus fluids and inotrope treatment. Acquired infections.
- Intraventricular haemorrhages
- Nil per mouth due to suspected necrotizing enterocolitis.
- Other conditions requiring transfer to higher level of care.

Of the above examples, death and any condition with risk of death and prolonged hospital care and future disability are categorized as severe adverse events.

A Data Safety Monitoring Board (DSMB) will oversee the study and its data independently. The DSMB will be chosen to include competence in neonatology, epidemiology, global health, and biostatistics. The DSMB will receive quarterly reports from a statistician on safety data. Based on this the DSMB can recommend pausing study enrollment, modify the study intervention or stop the study.

Investigator, co-investigators, and research nurses will within 24 hours report all serious adverse events to sponsor, and all adverse events on a monthly basis. The sponsor will inform the DSMB within one week of a serious event and collated every 6 months for adverse events.

An interim analysis is planned after 30 subjects have been enrolled.

10.4 Reporting of adverse events

10.4.1 Reporting of adverse events

All adverse events shall be reported on provided report form in the CRF.

10.4.2 Reporting of serious adverse events

Serious adverse events shall be reported to sponsor on assigned SAE form within 24 hours from researcher being aware of event. Complementary information that describes the outcome of the SAE and its ongoing management shall be reported as soon as available. The originals of these documents shall be filed in CRF and research folder.

10.5 Follow-up of adverse events

Since the intervention includes the first six hours of life, our study subjects will be receiving ongoing care then and for a considerable time thereafter. Adverse events are an expected part of care of preterm infants and will receive

the customary clinical care. Nevertheless, for all study subjects every adverse event and subsequent outcomes will be documented as described above.

11 STATISTICS AND DATA MANAGEMENT

11.1 Data management

Data will be collected manually on paper, using paper based CRF. At a later stage electronic CRF may be introduced. From these data will be fed into a database within one week, in English with translation from Swedish and Norwegian as required. In the CRF data will be anonymous, with a confidential study ID without sensitive personal identifiers. The study ID will be found in the hospital case record, as well as coded and kept securely with secure passwords. Investigator is responsible to ensure that the data entered into the CRF is correct and will attest in writing in each case.

Data to be entered into the CRF will be carefully defined before study launch, with a defined source code. All adverse events will be reported to sponsor and entered into CRF and database.

All handling of data will be according to the guidelines of Good Clinical Practice (GCP). Investigator is responsible for ensuring source data is accessible for monitoring.

All changes and corrections of the CRF must be dated, signed, and explained, and the originally entered data should be retained. When all data has been entered, verified, and validated it will be locked.

11.2 Statistiscal analysis

Primary outcome analysis will be done according to intention to treat (ITT) on all subjects that met inclusion criteria without exclusion criteria and were randomized. Per protocol analysis will also be undertaken based on actual adherence to allocated place of care. For safety measures the entire sample will be included. All continuous variables will be described with sample size and number of observations, average and median values, with standard deviations and minimum and maximum values. All categorical variables will be summarized in frequency tables. For the primary outcome a mixed model-regression analysis will be done to evaluate the effect of gestational age, birth order, single or multiple birth, intrauterine growth retardation, mode of birth (vaginal or caesarean), antenatal corticosteroids given or not, and birth weight. The null hypothesis is that there is no difference in the groups for the primary outcome. The analysis will be done for the whole study population as well as those that received the intervention.

An interim analysis will be done for the primary outcome on the first 30 patients enrolled.

Secondary outcomes that are continuous or categorical will be analyzed by linear or mixed model regression models, and dichotomous variables by logistic regression models. Stratification factors utilized in the randomization will be used as covariates. Qualitative and exploratory methods will be used as required.

11.3 Determination of sample size

Sample size calculations are based on the two studies on immediate SSC in low-birth-weight infants (6,7) where the proportion of maximum SCRIP score at 6 hours of age required 100 infants to reach 90% power with a 2 sided test, p=0,05. We expect a healthier cohort in Sweden and Norway, so we anticipate a smaller difference between the groups, and therefore plan to include 150 infants for the primary outcome of physiological stabilization. For one of the secondary outcomes, long term psychomotor development, a clinically significant difference of 0,3 SD on the Bayley test, with 80% power and a 2-sided test, p=0,05, the sample size is 104 patients, very similar to the primary

outcome. For many of the secondary outcomes there are no earlier studies to guide sample size calculation, therefore data will be collected on all possible subjects, or a subsample of what is possible in the circumstances.

12 ACCESS TO SOURCE DATA

Principal investigators have permission from the head of the clinical department to peruse and monitor hospital case records of enrolled infants and their mothers, and this will also be explained to parents in the informed consent interview.

13 QUALITY CONTROL

13.1 Source data

The major part of data collected is clinical in nature, which is found in the hospital case record, this is the primary data source. Investigator is responsible to ensure that the source of all data collected is recorded and can be retrieved. The cardiorespiratory parameters for the SCRIP score are collected more frequently than clinical routine and will therefore be collected on paper. This applies also to questionnaires and interview guide sheets. Various samples will be analyzed and results will come from different laboratories.

The subject's hospital case record will include a note as to the infant's participation in the study, the study name, the allocation arm, study ID number and contact persons for study.

13.2 Monitoring

The conduct of the study is described in this protocol, and in more detailed written form as Standard Operating Procedures (SOPs). Investigator is responsible for all collaborators contact details, areas of responsibility, availability of CV's, and the delegation list. In Sweden the trial will be monitored by the Karolinska Trial Alliance (KTA) and sponsor is responsible for establishing a monitoring plan. Monitoring in Norway will be done in a similar way, though monitor is not yet appointed.

14 ETHICS

14.1 Ethics review board

Ethical approval for this study has been granted by ethics boards in Stockholm, Sweden and Stavanger, Norway.

14.2 Ethical conduct of the study

The study will be conducted according to this protocol, and according to GCP, and according to the declaration of Helsinki. All investigators and research staff will have GCP training.

<u>Risk of hypothermia:</u>

A pilot study (10) suggested that infants of this gestational age may run the risk of hypothermia when cared for in SSC. To ensure that infants allocated to intervention stay warm they will be carefully dried before they are placed on parent's chest, and then covered with warmed textiles. The very smallest and those requiring longer stabilization may start on the resuscitaire where adequate warmth is ensured while ventilation or CPAP is initiated. Should any infant in SSC become hypothermic despite the described measures, it will be placed in a warm incubator and then return to SSC only when temperature is normal.

Sudden Unexpected Postnatal Collapse:
Sudden Unexpected Postnatal Collaps (SUPC) has recently been highlighted: seemingly healthy full-term infants may become suddenly lifeless and required resuscitation (11). SUPC has been associated with unobserved SSC and unsupervised breastfeeding in primiparous mothers, in prone position, and exhaustion with lack of attention in parents. In our study we do not see any risk of SUPC, as these infants will be maintained in a safe position with a garment and will be constantly observed by research staff and monitored by pulse oximetry.

Poorer observation:
Infants in SSC need to be covered by a blanket or garment to ensure they stay warm. This may result in the ward staff not having the same visual oversight of the infant as they would in an incubator or cot. In this study infants as described above will be monitored electronically, and the care of small and unstable infants already includes regular and frequent checks of the infant's condition and vital signs. In addition, as parent is always near the infant and can indicate if all is not well.

Discomfort:
The intervention has been observed to provide comfort to preterm infants. Ward staff will however need to provide comfort to the parents, including the reassurance that it is not the parents' responsibility to monitor their infants, the parents should feel secure and not abandoned knowing they are not alone with their newborn unstable premature baby. Ward staff may also feel that the control group infants are receiving substandard care, as they have experience in providing SSC to infants born with higher gestational age. Blood sample collections in the study have been timed to coincide with necessary routine and clinically indicated blood sampling, with the exception of the 3–4-month sample and the 2-year sample that do require extra needling. Buccal swabbing and skin swabbing may be slightly uncomfortable for the infant and may predispose to mild anxiety when repeated later in the study.

Confidentiality:
The research team and the monitors will access the patient case records as part of the study. The research nurse and the investigator that does the screening of mothers for enrollment will also be examining case records before mothers give consent. Once data has been collected it will be tagged with a study ID so that all subsequent analysis is totally anonymous.

14.3 Risk benefit evaluation

Infants in the control group will receive all care in accordance with current clinical best practice. Infants in the intervention arm may benefit from hypothesized advantages. All study subjects will receive additional follow-up compared to those not participating. This might be strenuous but can also provide additional comfort and reassurance that their infants are receiving extra care at the beginning of their lives and after discharge. It may provide them with increased opportunity to ask questions and an assurance that their infant has been fully investigated. If anything of significance is discovered during follow-up, immediate referral will provide early intervention support.

If we find positive short and or long-term benefits of the intervention, this will lead to a more equitable and evidence-based care than is currently provided. Should a potential risk be identified, this can be incorporated into care plans for better outcomes. On the other hand, if we find tangible risks with SSC provided to these very small and fragile infants, we will with this evidence base be able to stem the trend observed in many hospitals of giving more and more SSC to smaller and sicker babies without any evidence base of safety and effectiveness. There is also the risk that this form of care receives a tarnished reputation if not suitable for small and fragile infants, which

would make staff and parents afraid to provide SSC even on bigger and stable infants where the benefits are well established.

14.4 Trial subject information and consent

The research team (research nurse and PhD student) will screen antenatal and delivery ward admissions and communicate with clinical staff on potential preterm births. When a woman with threatening preterm birth is identified the information on the study will be provided verbally and in writing by the research team, but only after general information about preterm birth has been provided. When the parents (both, if present) have had opportunity to ask questions they will be invited to give consent, and subsequently sign consent forms. This will also be noted for the attention of neonatal ward staff, so that they are aware of this consent and potential research subject. The original consent form is archived, and the parents receive a copy.

15 DATA MANAGEMENT AND ARCHIVING

15.1 Case Report Forms

All data to be analyzed will be entered into a CRF, by the research nurse or by the PhD student, later the principal investigator will verify and sign. Should any changes be made, this should be signed off by the person making them, and a reason provided. Once all data is entered, verified, and signed off the file will be locked and cannot be changed further.

15.2 Archiving

All source data that is not in the infant's hospital case record will be archived in a fire and waterproof manner for ten years at the site of the study. Principal investigators are responsible to ensure this is done.

16 FINANCING AND INSURANCE

The head of the pediatric service in Stockholm and the health service head of Stavanger have approved the study. All patients in the study are therefore covered by health insurance as all other patients in the county of Stockholm and Stavanger.

17 PUBLICATION OF RESULTS

A publication plan has been initiated. A separate publication is planned for each outcome, and potentially some will also have short- and long-term outcome papers.

18 OTHER

18.1 Additions to the protocol

Any changes in this protocol will be done in collaboration with all study sites, principal investigators and sponsor, and if indicated after approval by ethics review boards.

18.2 Research staff information

Principal investigator takes responsibility to ensure that all study team members are fully informed and have adequate knowledge and competence for the conduct of their contribution to the study. Meetings will be conducted and training provided.

19 REFERENCES

1. Blencowe H, Lee AC, Cousens S, Bahalim A, Narwal R, Zhong N, et al. Preterm birth-associated neurodevelopmental impairment estimates at regional and global levels for 2010. Pediatr Res. 2013 Dec; 74 Suppl 1: 17-34

2. Moore ER, Anderson GC, Bergman N, Dowswell T. Early skin-to-skin contact for mothers and their healthy newborn infants. The Cochrane database of systematic reviews 2012; (5): CD003519.

3. Conde-Agudelo A, Diaz-Rossello JL. Kangaroo mother care to reduce morbidity and mortality in low birthweight infants. The Cochrane database of systematic reviews 2016; (8): CD002771.

4. Worku B, Kassie A. Kangaroo mother care: a randomized controlled trial on effectiveness of early kangaroo mother care for the low birthweight infants in Addis Ababa, Ethiopia. Journal of tropical pediatrics 2005; 51(2): 93-7.

5. Sankar MJ, Natarajan CK, Das RR, Agarwal R, Chandrasekaran A, Paul VK. When do newborns die? A systematic review of timing of overall and cause-specific neonatal deaths in developing countries. Journal of perinatology : official journal of the California Perinatal Association 2016; 36 Suppl 1: 1-11.

6. Bergman NJ, Linley LL, Fawcus SR. Randomized controlled trial of skin-to-skin contact from birth versus conventional incubator for physiological stabilization. Acta Paediatrica 2004;93(6):779-85.

7. Chi Luong K, Long Nguyen T, Huynh Thi DH, Carrara HP, Bergman NJ. Newly born low birthweight infants stabilise better in skin-to-skin contact than when separated from their mothers: a randomised controlled trial. Acta Paediatrica 2016; 105 (4): 381-90

8. WHO. WHO recommendations on interventions to improve preterm birth outcomes. Geneva: World Health Organization; 2015.

9. WHO. Kangaroo mother care: a practical guide; 2003.

10. Sundberg Bo, Thermoregulation and physiological stabilization by skin-to-skin contact at birth in preterm infants. Medical Degree project, Karolinska Institutet, 2016.

11. Pejovic N, Herlenius E. Unexpected collapse of healthy newborn infants: risk factors, supervision and hypothermia treatment. Acta Paediatrica 2013;102(7):680-8

20 SIGNATURES

Principal investigator at Karolinska (Danderyd, Huddinge och Solna)
Agnes Linnér, specialistläkare
PO Sjuka Nyfödda Barn, Inst för Kvinnors och Barns Hälsa
Karolinska Universitetssjukhuset

Signature Date

Principal investigator at Stavanger
Siren Rettedal, specialistläkare, PhD
Stavanger Universitetssykehus

Signature Date

Coordinating investigator (Danderyd, Huddinge och Solna)
Wibke Jonas, PhD
Inst för Kvinnors och Barns Hälsa
Karolinska Institutet

Signature Date

Sponsor
Björn Westrup, överläkare, PhD
PO Sjuka Nyfödda Barn, Inst för Kvinnors och Barns Hälsa
Karolinska Universitetssjukhuset

Signature Date
