# Supplementary material for: Skin-to-Skin Contact at Birth for Very Preterm Infants and Mother-Infant Interaction Quality at 4 Months: A Secondary Analysis of the IPISTOSS Randomized Clinical Trial
Source: JAMA Netw Open. 2023 Nov 30;6(11):e2344469. doi: 10.1001/jamanetworkopen.2023.44469 (PMC10690460; doi:10.1001/jamanetworkopen.2023.44469)
Supplement: Supplement 2. — eTable 1. Parent–Child Early Relational Assessment (PCERA) Items Assessed in a Free-Play Situation at 4 Months of Corrected Infant Age eTable 2. Comparison of Mother and Infant Characteristics of the Group From the IPISTOSS Study Included in the Parent–Child Early Relational Assessment Analysis at 4 Months of Corrected Infant Age (PCERA Group) and the Group That Did Not Participate in the 4-Month Follow-Up (Dropout Group) [file jamanetwopen-e2344469-s002.pdf]

Supplementary Online Content

Lilliesköld S, Lode-Kolz K, Rettedal S, et al. Skin-to-skin contact at birth for very preterm infants and mother-infant interaction quality at 4 months: a secondary analysis of the IPISTOSS randomized clinical trial. *JAMA Netw Open*. 2023;6(11):e2344469. doi:10.1001/jamanetworkopen.2023.44469

eTable 1. Parent–Child Early Relational Assessment (PCERA) Items Assessed in a Free-Play Situation at 4 Months of Corrected Infant Age

eTable 2. Comparison of Mother and Infant Characteristics of the Group From the IPISTOSS Study Included in the Parent–Child Early Relational Assessment Analysis at 4 Months of Corrected Infant Age (PCERA Group) and the Group That Did Not Participate in the 4-Month Follow-Up (Dropout Group)

This supplementary material has been provided by the authors to give readers additional information about their work.

© 2023 Lilliesköld S et al. *JAMA Network Open*.

eTable 1. Parent–Child Early Relational Assessment (PCERA) Items Assessed in a Free-Play Situation at 4 Months of Corrected Infant Age

PCERA subscales		Included items assessed	n	Cronbach's alpha
I.	Maternal Positive Affect, Sensitivity and Responsiveness (16 items)	Flat, unemotional, constricted voice; warm, kind tone of voice; positive affect; depressed mood; enthusiastic, animated, cheerful, "joie de vivre"; enjoyment, pleasure; quality and amount of positive physical contact; amount and quality of visual contact with child; amount of verbalization; quality of verbalizations; social initiative; contingent responsiveness to child's positive behavior; reads child's cues and responds sensitively and appropriate; connectedness; mirroring; resourcefulness, creativity	62	.89
II.	Maternal Negative Affect and Behavior (13 items)	Annoyed, angry, hostile voice; warm, kind tone of voice; expressed negative affect; irritable, frustrated, angry mood; displeasure, disapproval, criticism; quality and amount of negative physical contact; amount of verbalization; contingent responsiveness to child's negative behavior; structures and mediates environment; reads child's cues and responds sensitively and appropriate; flexibility/rigidity; intrusiveness; consistency, predictability	70	.86
III.	Infant Positive Affect, Communicative and Social Skills (11 items)	Expressed positive affect; happy, pleasant, content, cheerful mood; apathetic, withdrawn, depressed; alertness, interest; social behavior-initiates; social behavior-responds; quality of exploratory play; robustness; visual contact; communicative competence; readability	67	.87
IV.	Infant Dysregulation and Irritability (*8 items)	Expressed negative affect; anxious, tense, fearful; irritable/angry mood; emotional lability; avoiding, averting, resistance; attentional abilities; self-regulation, organizational capacities	69	.86
V.	Dyadic Emotional Tone, Reciprocity and Regulation (8 items)	Flat, empty, constricted tone of voice/facial expression; mutual enthusiasm, joyfulness, enjoyment, a sense of dyadic joie de vivre; reciprocity; frustrated, angry, hostile affect; tension, anxiety; joint attention, activity; organization, regulation of interactions; goodness of fit	71	.80

Scale compositions are based on the 4 month's feeding factors as recommended in the PCERA manual (Clark, 1985) that includes two parent scales, two infant scales and two dyadic scales. In this study, the two dyadic scales were combined into one global dyadic subscale.
*one item, "consolability/soothability", was dropped out in analysis due to scoring "non-ratable" in majority of observations (situation not happening)

© 2023 Lilliesköld S et al. *JAMA Network Open*.

eTable 2. Comparison of Mother and Infant Characteristics of the Group From the IPISTOSS Study Included in the Parent–Child Early Relational Assessment Analysis at 4 Months of Corrected Infant Age (PCERA Group) and the Group That Did Not Participate in the 4-Month Follow-Up (Dropout Group)

	PCERA group (*n* infants=71, mothers=56)	Drop-out group (*n* infants=20, mothers=17)	*P*-value
Gestational age, mean (SD), weeks	30.7 (1.3)	30.6 (1.4)	.82
Birthweight, mean (SD, range), grams	1535 (408, 555-2352)	1530 (367, 920-2440)	.96
Vaginal birth, n (%)	20 (36)	2 (12)	.06
Twins, n (%)	31 (44)	5 (25)	.13
Child sex, female, n (%)	29 (41)	11 (55)	.26
Preeclampsia, n (%)	18 (32)	8 (47)	.26
Primiparous, n (%)	32 (57)	11 (61)	.77
Maternal age, mean (SD, range)	32 (4.9, 21-45)	30 (4.6, 23-39)	.18
Cohabitant parents, n (%)	54 (96)	14 (82)	.53
University education mother, n (%)	39 (70)	9 (53)	.11
Mental health diagnosis mother, n (%)	8 (14)	1 (6)	.35
EPDS, Depressive symptoms, mean (SD, range)<br>One week post birth<br>Follow-up visit at fullterm	 11.4 (6.3, 0-25) *n*=52<br>6.8 (5.5, 0-20) *n*=43	 9.5 (4.7, 0-18) *n*=11<br>5.3 (2.9, 0-9) *n*=9	 .35<br>.27
STAI, Anxiety symptoms, mean (SD, range)<br>One week post birth<br>Follow-up visit at fullterm	 44 (12.8, 24-79) *n*=47<br>35.5 (9.8, 21-60) *n*=41	 35.6 (13.5, 21-60) *n*=9<br>30.4 (5.7, 21-39) *n*=8	 .08<br>.16

© 2023 Lilliesköld S et al. *JAMA Network Open*.
