# Supplementary material for: Skin-to-Skin Contact at Birth for Very Preterm Infants and Mother-Infant Interaction Quality at 4 Months: A Secondary Analysis of the IPISTOSS Randomized Clinical Trial
Source: JAMA Netw Open. 2023 Nov 30;6(11):e2344469. doi: 10.1001/jamanetworkopen.2023.44469 (PMC10690460; doi:10.1001/jamanetworkopen.2023.44469)
Supplement: Supplement 3. — Data Sharing Statement [file jamanetwopen-e2344469-s003.pdf]

Data Sharing Statement

Lilliesköld. Skin-to-Skin Contact at Birth for Very Preterm Infants and Mother-Infant Interaction Quality at 4 Months. *JAMA Netw Open*. Published November 30, 2023.
doi:10.1001/jamanetworkopen.2023.44469

Data
Data available: No

Additional Information
Explanation for why data not available: When obtaining consent from the parents included in the study we have not specifically asked for their permission to share data to the larger scientific community.
